# The influence of preoperative knee flexion contracture severity on short-term outcome of orthopedic surgery in ambulatory children with bilateral cerebral palsy

**DOI:** 10.1186/s12891-021-04362-x

**Published:** 2021-05-25

**Authors:** Evelina Pantzar-Castilla, Brian Po-Jung Chen, Freeman Miller, Jacques Riad

**Affiliations:** 1 Department of Orthopedics, Örebro University Hospital, Södra Grev Rosengatan, Örebro, Sweden; 2 Department of Orthopedic Surgery, Nemours/AIfred I. duPont Hospital for Children, Wilmington, Delaware, USA; 3 Department of Pediatric Orthopedics, Chang Gung Memorial Hospital, Taoyuan, Taiwan; 4 Department of Orthopedics, Institute of Clinical Science, Sahlgrenska Academy, University of Gothenburg, Gothenburg, Sweden; 5 Department of Orthopedics, Skaraborg Hospital Skövde, Skövde, Sweden

**Keywords:** Cerebral palsy, Knee flexion contracture, Gait deviation index, Gait analysis, Orthopedic surgery

## Abstract

**Background:**

Indications and cutoff value of deformities to determine surgical procedures for flexed knee gait are not clear. The aim was to determine the influence of none or mild, and moderate preoperative knee flexion contracture on the improvement of gait after orthopedic surgery in children with bilateral cerebral palsy (CP).

**Methods:**

Inclusion criteria; bilateral CP, Gross Motor Function Classification System level I-III, and pre- and post operative-gait analysis. The 132 individuals identified were categorized into 2 groups based on the severity of knee flexion contracture (group 1: none or less than 11°; group 2: greater than or equal to 11°), and then matched according to the exact same soft tissue and/or bony orthopedic surgical procedures performed. The indication for surgery was to prevent progressive development of knee flexion contracture and stance phase flexed knee gait. Pre- and postoperative physical examination and gait analysis data were analyzed retrospectively.

**Results:**

Sixty (30 + 30) children, with mean age 10.6 years in each group, were included. The average follow-up time was 17 months.

Gait Deviation Index (GDI) improved in group 1 from mean 66 (SD 19) to 74 (15), *p* = 0.004, and in group 2 from 60 (13) to 69 (15), *p* = 0.001. Knee flexion in stance improved in group 1 from 21.4 (16.1) to 12.1 (16.0) degrees, *p* = 0.002, and in group 2 from 32.2 (14.2) to 17.0 (15.9), *p* = 0.001. Step length improved in both groups, *p* = 0.017 and *p* = 0.008, respectively.

Only in group 2 significant improvement was noted in walking speed, *p* = 0.018 and standing function, Gross Motor Function Measure (GMFM-D), *p* = 0.001. Knee flexion contracture decreased in group 1 from mean 4.6 (5.3) to 2.1 (8.3) degrees, *p* = 0.071 and in group 2 from 17.2 (4.9) to 9.6 (9.3), *p* = 0.001.

There was no statistical difference between groups in pre-post improvement of GDI or other variables, except GMFM-D.

**Conclusions:**

Relative mild to moderate preoperative knee flexion contracture does not influence the short-term improvement of gait after orthopedic surgery in children with bilateral CP.

## Background

Walking with excessive knee flexion in stance phase, i.e., crouch gait, is a common gait pattern in children with bilateral cerebral palsy (CP) [1, 2]. Studies show that Gross Motor Function Classification System (GMFCS) level, age, muscle strength, and motor control are associated with flexed knee gait [3–6]. Hamstring muscle over activity and shortening may cause flexed knee gait [3, 7]. Over lengthening of the gastroc-soleus muscle complex was shown to be a common cause of severe crouch [8]. Skeletal deformities, including malalignment, excessive femoral internal rotation, external tibial torsion and pes planovalgus with midfoot break, causing lever arm dysfunction, may also contribute to flexed knee gait [9–11].

During growth, especially in adolescence, knee flexion contracture may develop with increased knee flexion in gait [9, 11]. Flexed knee gait does not typically cause severe problems in the young child; however, with increased weight and height, progresses and ultimately limits walking ability [12].

Surgical treatment for flexed knee gait with more or less knee flexion contracture is performed at multiple levels [2, 13–17]. Common procedures include hamstring muscle lengthening, rectus femoris transfer, posterior knee capsulotomy (PKC) and distal femoral extension osteotomy (DFEO), combined with patellar tendon advancement (PTA) or patellar tendon shortening (PTS) [2, 13–17]. In addition, surgical correction of malalignment and foot deformity, are also performed to improve knee extension [9, 10, 18].

The exact indications and severity of deformities for surgical treatment addressing flexed knee gait, with or without knee flexion contracture are still debated [19]. Several studies focus on different surgical procedures directed towards knee flexion contracture per se, and do not necessarily discuss the other surgical procedures performed at the same time. Furthermore, the cutoff value of knee flexion contracture when posterior knee capsule release or distal femoral extension osteotomy, with patellar tendon duplication or advancement, is warranted is varying [18, 20–22]. From a biomechanical point of view, restoring lever-arm function depending on malalignment is a prerequisite for knee extension [18, 23]. Proximal femur rotational osteotomy is performed to align the knee axis. Increased tibial external rotation may need correction. In addition, a stable, plantigrade foot with forward foot progression is an important part of restoring alignment and lever-arm function. Correction of the often-severe pes planovalgus foot with midfoot break, in providing an effective plantar flexion knee extension couple [10, 18].

Hence the significance of knee flexion contracture and how it may influence the outcome of different orthopedic surgical procedures to improve and prevent the progression of flexed knee gait, is not clear and needs to be further studied.

The aim of this study was to determine the influence of preoperative, none or mild knee flexion contracture with moderate knee flexion contracture, in matched surgical groups, on the improvement of gait in ambulatory children with bilateral CP.

## Methods

From the Epic medical records database (Epic Systems Corporation, Verona, WI, USA), 132 children with bilateral CP who underwent orthopedic surgery, with both pre and postoperative three-dimensional gait analysis between 2004 and 2015 were identified retrospectively. All gait analysis assessments were performed at the same gait laboratory. The inclusion criteria were: diagnosed as bilateral CP; GMFCS level I to III at the preoperative visit; preoperative gait analysis performed within 12 months before surgery; and postoperative gait analysis between 10 and 30 months after surgery. The children were categorized into two groups based on the preoperative severity of knee flexion contracture. Group 1 comprised those with none or less than 11° of knee flexion contracture; group 2 comprised  those with knee flexion contracture greater than or equal to 11°. In addition, the two groups were matched according to orthopedic surgical procedures performed. When the exact same set of procedures was noted in one child from group 1, with another child in group 2, they were considered matched, which resulted in 30 + 30 children. The surgical procedures performed are listed in Table 1. The remaining 72 children could not be matched. All methods used in this study were carried out in accordance with relevant guidelines and regulations.

**Table 1:** Break down of orthopedic surgical procedures in group 1 and 2

Surgical procedures	Group 1 (*n* = 30)	Group 2 (*n* = 30)
HS	3	3
HS / Calf	1	1
HS / Rectus	2	2
HS / Add	2	2
HS / Calf / Add	3	3
HS / Calf / Rectus / Add	1	1
HS / Calf / Add / Iliopsoas	1	1
HS / Foot	2	2
HS / Calf / Tibia	2	2
HS / Calf / Femur	1	1
HS / Knee capsule / Foot	1	1
HS / Rectus / Foot	2	2
HS / Add / Foot	1	1
HS / Calf / Rectus / Tibia	1	1
HS / Calf / Rectus / Femur	1	1
HS / Calf / Tibia / Femur	1	1
HS / Rectus / Foot / Tibia	2	2
HS / Rectus / Foot / Tibia / Femur	3	3

HS: hamstring lengthening (semitendinosus transection and semimembranosus circumferential incision of the fascia at two levels and sometimes the corresponding fascia incisions of the biceps femoris to achieve at least 30–40 degrees of popliteal angle perioperative); Calf: calf muscle lengthening (gastrocnemius lengthening/resection was performed sometimes with an additional incision of the soleus fascia with the goal of achieving a plantar foot with the knee extended and care taken not to over lengthening); Add: hip adductor lengthening; Rectus: rectus femoris transfer; Tibia: tibia derotational osteotomy; Femur: femoral derotational osteotomy; Knee capsule: posterior knee capsule release. Iliopsoas: iliopsoas lengthening. Foot: foot correction with calcaneal lengthening or subtalar fusion, and a medial foot column correction/stabilization

### Gait analysis

Three-dimensional gait analysis was performed with an eight-camera motion analysis system (Motion Analysis Corporation, Rohnert Park, CA, USA) to obtain kinematic gait data. The markers were attached to specific anatomical landmarks in accordance with a modified Helen-Hayes model [24, 25]. The change of Gait Deviation Index (GDI) before and after surgery was calculated and used as the primary outcome of this study [26]. GDI describes the deviation of gait kinematics from the normal population. Walking speed and step length were also calculated. The use of assistive devices during the gait analysis was documented.

### Physical examination

**Fig. 1:**
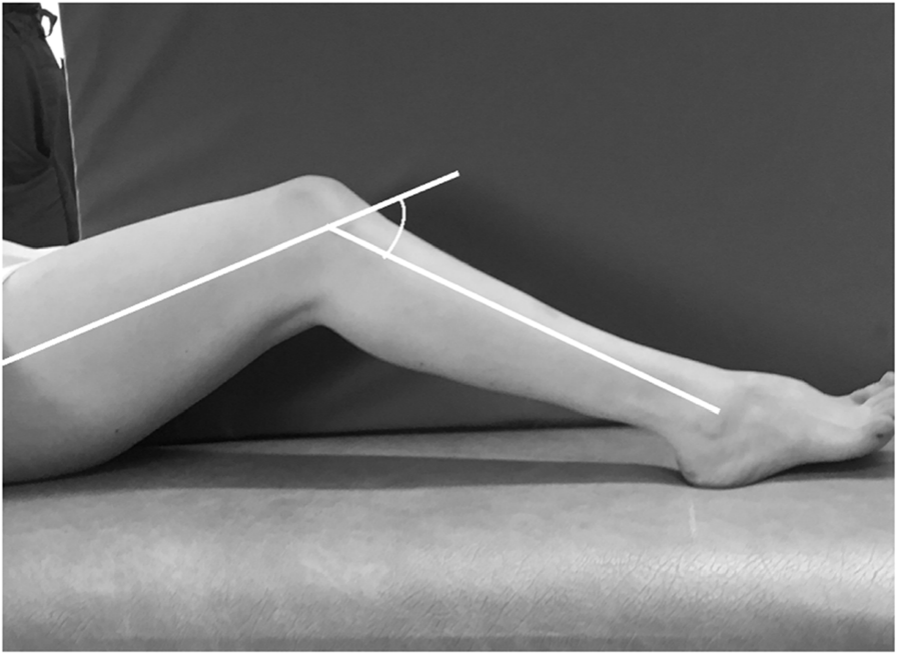
Examination position of knee flexion contracture. By using a goniometer on to the lateral side of the thigh and shank

The physical examination was performed at both pre and postoperative gait analysis visits. Knee flexion contracture was defined as not fully extending the knee joint to neutral position (i.e., 0° of knee extension). It was measured, while the child supine, with the hip in full extension and the ankle in plantarflexion, by a goniometer on to the lateral side of the thigh and shank. The hip was always in maximum extension to measure the knee flexion contracture to avoid the influence of the hamstring contracture. Figure 1. The inter-rater reliability for passive knee extension (knee flexion contracture) was 3.5 degrees_._ The popliteal angle was measured supine with the hip flexed to 90 degrees, and the knee was extended until the pelvis starts to rotate and at this position, the goniometer measurement was assessed. The hamstring muscle spasticity is measured by the modified Ashworth Scale, a 6-point rating scale for the level of muscle resistance to passive movement [27]. The Gross Motor Function Measure, dimension D (GMFM-D), which is a functional assessment of standing ability developed for children with CP, was evaluated as well as the GMFCS level [6, 28].

No difference was found in knee flexion contracture (*p* = 0.375) between the right side (11.0° [SD 8.2]) and left side (10.4° [SD 8.6]) on preoperative physical examination. Results from the right side were used as the representative data of each individual except GMFM-D, which evaluates the bilateral standing function.

### Statistics

Graphical exploration with frequency histograms and standard plots was performed to evaluate normal distribution of collected data. Since the data were normally distributed, parametric tests were used. Pearson’s correlation coefficient to study the association of different variables with knee flexion contracture and paired-sample t-tests and independent-sample t-tests to compare groups. A *p*-value less than 0.05 was regarded as statistically significant. All statistical analyses were performed in SPSS, version 22 (IBM Corp., Armonk, NY, USA).

## Results

Based on the severity of knee contracture (Group 1: none or less than 11°; Group 2: greater than or equal to 11°) and matching with surgical procedures performed, 60 individuals (*n* = 30 in each group) were included in the final analysis. Break down of detailed surgical procedures (Table 1), demographics, GMFCS level, the use of assistive devices, and follow-up time from surgery to postoperative gait analysis were documented (Table 2). Age, height, and weight were not significantly different between groups. Higher GMFCS levels and more assistive devices were found in group 2 compared to group 1. The follow-up time was longer in group 2 (*p* = 0.020).

**Table 2:** Demographics of children in group 1 and 2

	Group 1 (*n* = 30)	Group 2 (*n* = 30)
**Age of surgery (years)** mean (range)	10.6 (3.6–17.1)	10.6 (4.9–18.4)
**Gender** male / female	12 / 18	21 / 9
**Height (cm)** mean (range)	132.3 (89.0–169.0)	129.8 (95.0–179.0)
**Weight (kg)** mean (range)	35.1 (11.2–71.5)	34.0 (13.3–68.0)
**GMFCS level** I / II / III	5 / 14 / 11	2 / 10 / 18
**Using assistive devices** n (%)	12 (40.0%)	17 (56.7%)
**Follow-up (months)** mean (range)	16 (10–28)	18 (11–32)

In both groups, significant improvement was found for GDI, knee kinematics, step length, popliteal angle and hamstring muscle spasticity. However, significant improvement of walking speed and GMFM-D were found only in group 2 (*p* = 0.018 and *p* = 0.001, respectively), but not in group 1 (*p* = 0.786 and *p* = 0.553, respectively). Knee flexion contracture decreased in group 1 (*p* = 0.071), but not as significantly as in group 2 (*p* = 0.001) (Table 3). There was no statistical difference in the pre-post improvement of GDI or other variables between groups, except for GMFM-D (Table 3).

**Table 3:** Pre and post-operative data in group 1 and 2 and the changes between the groups

	Group 1 (*n* = 30)				Group 2 (*n* = 30)				Group 1 vs Group 2	
	Pre Mean (SD)	Post Mean (SD)	Mean diff (SD)	***P-*** value	Pre Mean (SD)	Post Mean (SD)	Mean diff (SD)	***P-*** value	Mean diff (95%CI)	***P-*** ***value***
Knee flexion contracture (degrees)	4.6 (5.3)	2.1 (8.3)	2.5 (7.4)	0.071	17.2 (4.9)	9.6 (9.3)	8.2 (8.8)	0.001*	5.7 (1.5–9.9)	0.009*
Gait Deviation Index	66 (19)	74 (15)	8 (13)	0.004*	60 (13)	69 (15)	9 (12)	0.001*	1 (5–8)	0.675
Knee flexion at initial contact (degrees)	47.6 (13.4) range 30–91	39.1 (12.1) range 12–69	8.5 (13.4)	0.002*	52.2 (9.4) range 30–72	42.4 (10.4) range 32–75	9.8 (13.0)	0.001*	1.3 (−5.6–8.1)	0.716
Minimal knee flexion in stance (degrees)	21.4 (16.1) range 21–75	12.1 (16.0) range 12–61	9.3 (14.7)	0.002*	32.2 (14.2) range 18–69	17.0 (15.9) range 8–74	15.2 (16.3)	0.001*	5,9 (− 2.1–13.9)	0.148
Walking speed (cm/s)	79 (27)	78 (30)	−0.3 (22,8)	0.786	55 (30)	67 (35)	11.5 (25.1)	0.018*	−11,8 (0.6–24.2)	0.062
Step length (cm)	40.8 (10.0)	43.9 (10.0)	3.1 (6.4)	0.017*	33.9 (13.2)	39.3 (14.9)	5.4 (10.5)	0.008*	−2.3 (−2.3–7.0)	0.316
GMFM-D (points)	23.3 (8.0)	24.2 (9.5)	−1.9 (8.2)	0.553	16.5 (10.1)	22.1 (9.3)	3.1 (6.7)	0.001*	−5.0 (1.1–8.8)	0.014*
Popliteal angle (degrees)	59.0 (12.4)	49.1 (17.0)	9.9 (15.1)	0.001*	71.9 (12.2)	55.7 (13.0)	16.2 (18.5)	< 0.05*	6.2 (−2.6–15.1)	0.163
Hamstring spasticity (points)	1.3 (0.8)	0.9 (0.5)	0.4 (0.8)	0.013*	1.6 (0.7)	0.9 (0.5)	0.7 (0.8)	< 0.05*	0.2 (−0.2–0.6)	0.358

Pre: pre-operative; Post: post-operative; SD: standard deviation; Mean diff: Mean difference; Gait Deviation Index; GMFM-D: Gross Motor Function Measure, dimension D; *: statistically significant. Knee flexion at initial contact refers to the start of the gait cycle when the foot first has contact with the floor. Minimal knee flexion in stance refers to the point when the least (minimal) knee flexion occurs

## Discussion

The aim of this study was to evaluate the influence of preoperative knee flexion contracture on the improvement of gait after orthopedic surgery in ambulatory children with bilateral CP.

By comparing pre and postoperative data retrospectively between matched groups (*n* = 30 in each group) in whom the same set of orthopedic surgical procedures was performed but had different severity of pre-operative knee flexion contracture, we found that the severity of knee flexion contracture did not directly influence the degree of improvement of postoperative gait pattern. The same improvement was found in gait kinematics, i.e., GDI, and step length despite the severity of knee flexion contracture. In addition, popliteal angle improved, and hamstring muscle spasticity deceased in both groups. Not surprisingly, individuals with more severe knee flexion contracture (group 2) had lower gross motor function categorized by GMFCS when compared with those with less severe knee flexion contracture (group 1) as previous studies showed [3, 5].

The overall indication for surgery in this relatively young population was to improve and prevent the progress of flexed knee gait.

The surgery was not primarily directed towards knee flexion contracture nor at a specific surgical procedure, but more generally, to treat flexed knee gait by correction of short muscles and tendons, and correction of malalignment including foot deformity, with all of the additional procedures in the SEMLS, in this surgically matched cohorts. The conceptual surgical planning for this approach is to prevent progressive development of knee flexion contractures and stance phase flexed knee gait. This was performed by lengthen short muscles, transfer the rectus femoris if reduced and delayed knee flexion in swing. Bony surgery including femoral and tibial osteotomies and foot correction was performed to correct malalignment. The aim was to restore the knee axis, foot stability and foot progression to achieve a more effective plantar-flexion knee extension couple to improve knee extension in stance phase [9, 10, 18]. Naturally, the extent of the surgical correction performed depended on the degree of deformity. All children walked with increased knee flexion and had at least hamstring lengthening performed with effect on stance phase knee function with improved knee extension at initial contact and in mid-stance. (Table 3).

The cutoff value of knee flexion contracture was set at 11° for categorizing group 1 and group 2 in order to make comparisons with previous studies, where cutoffs between 10 and 15 degrees have been reported [14, 22]. We also found the cutoff reasonable in our relatively young cohort that had not developed more severe knee flexion contracture. Boyer et al. reported similar improvement to our results in their long-term follow-up after DFEO with PTA on a cohort with a comparable degree of preoperative knee flexion contracture as group 2 in our study [14].

Taylor et al. reported treatment with PKC for knee flexion contracture of 21°, and DFEO for more severe knee flexion contracture of 34° [17]. Their results showed improvement in both dynamic and static knee flexion contracture after PKC and DFEO. They considered PKC as part of multilevel surgery to be a better option than DFEO for those who had less severe knee flexion contractures. Interestingly, they reported more improvement of knee flexion contracture in the DFEO group than in our group 2 (26° vs. 8°). Moreover, their DFEO group had more pronounced preoperative knee flexion contracture (34° vs. 17°). Ma et al. reported the outcome of combined hamstring lengthening and transfer for a preoperative knee flexion contracture greater than 15° [22]. They considered the procedure as part of multilevel surgery as in our study, but excluded any osteotomies or foot surgeries. Their two-year follow-up showed improvement of knee flexion in stance phase from 17° to 3°.

Klotz et al. and Sossai et al. both used a cutoff value for knee flexion contracture of 10° or more to choose DFEO [20, 21]. The treatment algorithm at our center includes careful assessment and correction of rotational malalignment, including the femur, tibia and foot, in addition to sagittal plane deformities, to improve knee extension. In the study by Klotz et al., no torsional correction was mentioned. In our study group, the thorough foot correction of the frequently severe pes plan valgus deformity with mid-foot break included calcaneal lengthening or subtalar fusion, combined with medial mid-foot correction (i.e., flexion arthrodesis of the navicular-medial cuneiform joint), and calf muscle lengthening if needed.

Adolfsen et al. reported multilevel soft tissue surgery for flexed knee gait with mild preoperative knee flexion contracture as our group 1, and noted similar improvement [13]. Sossai et al. reported the same improvement of flexed knee gait in a cohort of 24 individuals (age range of 10–40 years) after PTS for those with no knee flexion contracture and after PTS with DFEO for those with more than 10° [20].

Knee flexion in stance phase improved in both of our groups, as in the studies by Taylor et al. and Boyer et al. [14, 17]. However, Taylor et al. showed a greater improvement in their DFEO group, from 59° (SD 16) to 25° (SD 25), compared to our results [17]. In addition, Taylor et al. with the more severe knee flexion contracture cohort found a greater improvement in GDI compared to our results, from 56 (SD 13) to 65 (SD 11) points in the PKC group and from 44 points (SD 12) to 67 points (SD 14) in the DFEO group [17].

Although GDI was shown to be improved in the study by Taylor et al., they found a decline in the gross motor function assessed by GMFM-D and decreased walking speed for both PKC and DFEO groups [17]. Interestingly, our results showed a significant improvement in both GMFM-D (5.6) and walking speed (12 cm/s) in group 2, but no significant change in group 1.

The limited improvement of function, GMFM-D in this study, and the modest or non-significant relation to improvement of kinematics gait variables has been previously reported with the Gillette Functional Assessment Questionnaire [29]. Functional Mobility Scale (FMS) was used by Ma et al. as their outcome measure besides kinematics [22, 30]. We did not collect FMS routinely for all individuals and hence the GMFM-D was chosen as the primary gross motor function outcome. We noted increased step length in both groups, although not significant.

In this short-term follow-up study, participants were matched based on surgical procedures performed to evaluate the impact of knee flexion contracture on surgical outcome using GDI, rather than studying the effect of a particular surgical procedure as in the study by Boyer et al. In their report, surgical procedures other than DFEO with PTA were not stated therefore making comparisons of the effectiveness of surgery difficult [14].

The indication when soft tissue and/or bony surgery is warranted, based on the degree of concurrent knee flexion contracture is not clear. Hamstring lengthening was performed in all individuals in our study (Table 1). Ma et al. pointed out the overall importance and effect of hamstring lengthening for improvement of knee stance-phase kinematics, and the possible additional benefit of hamstring transfer [22]. Since surgical procedures directed towards knee flexion contracture, with posterior capsule release or distal femoral extension osteotomy, and patellar tendon advancement is relatively extensive surgery, with quite cumbersome immobilization and long rehabilitation, these procedures should be used when there is a clear indication. In this study we have with the surgical treatment algorithm for flexed knee gait, found that the same improvement in gait pattern can be expected regardless if the child has mild or moderate knee flexion contracture.

As a retrospective study, it has inherent limitations. We had relatively small study group (30 + 30) children. In addition, the follow-up time was short (< 3 years). A longer follow-up time should be investigated in future studies to understand the long-term outcome predictively of the preoperative knee flexion contracture. Moreover, details of each child’s previous interventions were not provided since it is nearly impossible to obtain with high accuracy. However, the children were all assessed and treated at the same medical center according to the same algorithm of one senior orthopedic surgeon. The exact post-operative care, time of casting, degree and time of weight bearing, and the amount and physiotherapy program performed is not provided. Furthermore, the groups could not be perfectly matched according to GMFCS level and we had not included other variables that could influenced the composition of the two groups and the result such as, pre- and post-operative training, availability of orthosis and assistive devices. Possible cognitive, behavioral disabilities and co-morbidities i.e. seizures, could also influence and make the comparisons of groups difficult. Nevertheless, the groups were relatively comparable regarding age, height, weight and follow-up time, and fairly good regarding gender and using assistive devices (Table 2).

## Conclusion

In children with bilateral CP who had relatively mild to moderate preoperative knee flexion contracture, the severity of knee flexion contracture does not directly influence the short-term improvement of gait pattern after soft tissue and/or bony orthopedic surgery.

Our results indicate that although physical examination of passive-range-of motion is an important and easily accessible variable, the severity of knee flexion contracture does not necessarily determine the indication for specific orthopedic surgical procedures. A comprehensive multi-level assessment in the surgical treatment plan is most likely warranted.

## Data Availability

The datasets used and/or analyzed during the current study are available from the corresponding author on reasonable request.

## Abbreviations

CP: Cerebral palsy
GMFCS: Gross motor function classification system
GDI: Gait Deviation Index
GMFM-D: Gross Motor Function Measure, dimension D
PKC: Posterior knee capsulotomy
DFEO: Distal femoral extension osteotomy
PTA: Patellar tendon advancement
PTS: Patellar tendon shortening
FMS: Functional Mobility Scale
